# Implicating factors in the increase in cases of central precocious puberty (CPP) during the COVID-19 pandemic: Experience of a tertiary centre of pediatric endocrinology and review of the literature

**DOI:** 10.3389/fendo.2022.1032914

**Published:** 2022-11-30

**Authors:** C. Barberi, V. Di Natale, V. Assirelli, L. Bernardini, E. Candela, A. Cassio

**Affiliations:** ^1^ Department of Medical and Surgical Sciences, Istituto di Ricovero e Cura a Carattere Scientifico (IRCCS) Azienda Ospedaliera Universitaria di Bologna, Bologna, Italy; ^2^ Istituto di Ricovero e Cura a Carattere Scientifico IRCCS Azienda Ospedaliera Universitaria di Bologna, Program of Endocrine Metabolic Diseases, Unit of Pediatrics, Bologna, Italy; ^3^ Department of Medical and Surgical Sciences, Pediatric School of University of Bologna, University of Bologna, Bologna, Italy; ^4^ Department of Medical and Surgical Sciences, University of Bologna, IRCCS Azienda Ospedaliera Universitaria di Bologna, Program of Endocrine Metabolic Diseases, Bologna, Italy

**Keywords:** central precious puberty, COVID - 19, early puberty, thelarche premature, electromagnetic field, BMI - body mass index, endometrial rhyme, isolated pubarche

## Abstract

Sexual development is a complex mechanism activated by the hypothalamic-pituitary-gonadal axis. Over the last one hundred years there has been a decline in the age at puberty onset in industrialised countries. Some Italian studies showed an increase in diagnoses of Central Precocious Puberty (CPP) during the COVID-19 pandemic. It is thus supposed that in this period there was an increased impact of factors that can influence pubertal development. Our retrospective monocentric study aimed to confirm the existence of this phenomenon and analysed possible related factors. We retrospectively evaluated clinical, laboratory, radiological and ultrasound (US) data of 154 girls referred to our Tertiary Centre of Paediatric Endocrinology from January 2019 to April 2021 for different forms of Precocious Puberty. We subdivided the cases into subgroups according to the final diagnosis: CPP, Early Puberty (EP), isolated thelarche and isolated pubarche. The observation period was subdivided into: Period 1, before lockdown (1 January 2019 – 8 March 2020) and Period 2, lockdown and the following months (9 March 2020 – 30 April 2021). Period 2 was further divided into “restrictive lockdown period” (Period 2.1) (March 2020 – 14 June 2020, in which the schools were closed) and “less restrictive lockdown period” (Period 2.2) (15 June 2020 – 30 April 2021). We analysed data regarding the use of electronic devices before and during lockdown in a group of girls with CPP diagnosed in Period 2 and we compared the data with that of a control group. Our data show an increase in the number of new diagnoses of CPP during lockdown and in the following months, compared with the previous period. We also detected a higher use of PCs and smartphones in girls with CPP diagnosed in Period 2, compared with the control group. The percentage of the presence of endometrial rhyme detected during the pelvic ultrasound was higher in girls with CPP in Period 2, compared with the previous period. Based on our data we assume there was an environmental effect on pubertal timing that calls our attention to factors such as food, use of electronic devices and stress. We will need further studies to better understand this data.

## Introduction

The complex mechanism that leads to sexual development starts from the activation of the hypothalamic-pituitary-gonadal axis by an unknown primary input. Over the last one hundred years there has been a decrease in age at puberty onset in industrialised countries; menarche appears on average in girls about 12 and a half years old, and thelarche in girls between 9 and a half and ten years old (1, 2). The basis of this decline involves not only genetic factors (2, 3) but also environmental factors such as BMI (4), dietary habits, physical activity, stressors (5) and exposure to ECDs and electromagnetic fields (6, 7).

In Italy, the restrictions due to the coronavirus pandemic led to deep changes in people’s habits. Italians had to stay at home for several months. The schools closure and the beginning of distance learning led to an increase in the use of electronic devices. These devices were also used more frequently for extracurricular reasons (video games, TV, PC games) that resulted in an accumulation of hours spent in front of screens.

Forced home - staying at home also led a reduction in physical activities and an increase in consumption of junk food resulting in body weight gain.

In addition, the pandemic situation led to a growth in stressors for many families (fear of illness, fear for the health of loved ones, economic problems…).

Some previous Italian studies reported an increase in the number of new cases of Central Precocious Puberty (CPP) in girls during the COVID-19 pandemic (8–11) It has been assumed that during this period, the impact of factors interfering with pubertal development increased.

This retrospective monocentric study aimed to confirm the existence of this phenomenon and analysed possible related factors.

## Materials and methods

We retrospectively evaluated clinical, laboratory, radiological and ultrasound (US) data on 154 girls referred to our Tertiary Centre of Paediatric Endocrinology from January 2019 to April 2021 for different forms of Precocious Puberty. We subdivided the cases into subgroups according to the final diagnosis: CPP, Early Puberty (EP), isolated thelarche and isolated pubarche. CPP was defined according to Consensus Guidelines (12). Early puberty was defined as the onset of pubertal signs between 8 and 9 years old (13).

The observation period was subdivided into: Period 1, before lockdown (1 January 2019 – 8 March 2020), and Period 2, lockdown and the following months (9 March 2020 – 30 April 2021). Period 2 was further divided into “restrictive lockdown period” (Period 2.1) (9 March 2020 – 14 June 2020, in which the schools were closed) and “less restrictive lockdown period” (Period 2.2) (15 June 2020 – 30 April 2021).

**Figure 1:**
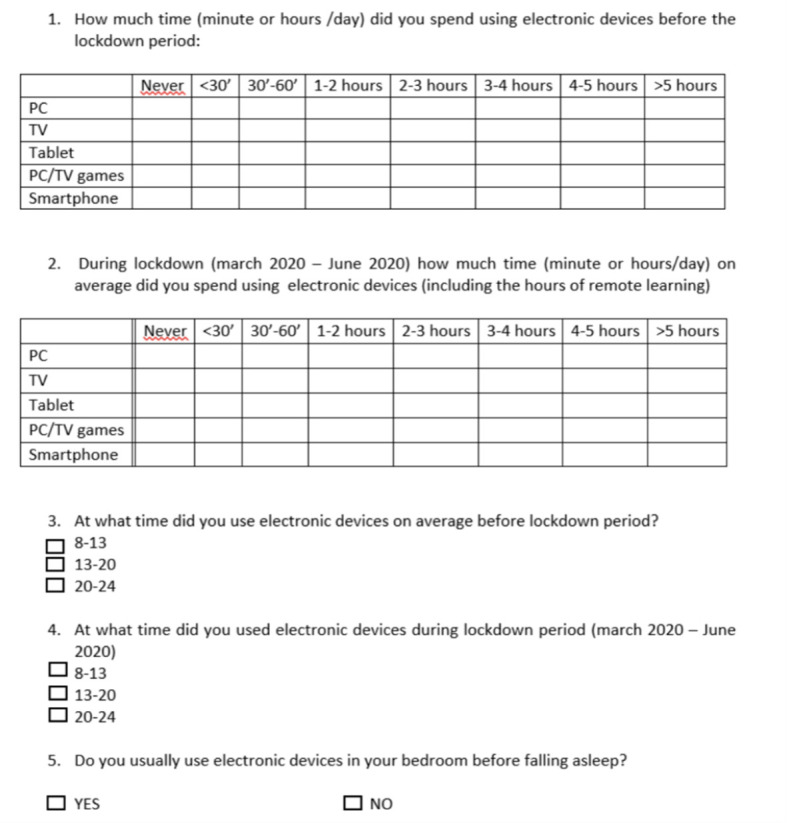
Questionnaire regarding the use of electronic devices.

We analysed data regarding the use of electronic devices obtained through a questionnaire (**Figure 1**) administered to 17 girls diagnosed with CPP in Period 2 and to 26 “short normal” controls matched for sex and age referred to our Centre in the same period.

We excluded girls with CPP associated with hypothalamic–pituitary congenital malformations, neurological, neurosurgical and/or genetic diseases, psychomotor delay, oncological diseases. We also excluded adopted girls with CPP.

Age, sex, ethnicity, family history of precocious and EP, mid-parental height, age at the onset of pubertal signs, and age at first observation were recovered from clinical records. Height, weight, birth weight and BMI were expressed as a standard deviation score (SDS) according to Italian standards (14). Pubertal stage was recorded according to Marshall and Tanner’s genital stage (15); we analysed the rate of progression of pubertal signs. We defined “rapid progression” as a transition from B2 to B3 in less than six months.

When available, we recorded levels at baseline of follicle-stimulating hormone [FSH], luteinizing hormone [LH], estradiol [E2]. A Gonadotropin-releasing hormone (GnRH) test was performed by i.v. administration of GnRH to 94 girls (37 in Period 1 and 57 in Period 2) with LH and FSH measurement at 0, + 30 and + 60 min.

A basal level of LH > 0,3 IU/L and/or a peak response of LH after GnRH infusion > 5 IU/L, with or without serum estradiol levels > pg/ml, were considered suggestive of CPP (16–18).

We estimated bone age (BA) from an X-ray of the left hand and wrist using the Greulich & Pyle atlas (19). Bone age advancement was defined as the difference between BA and chronological age expressed in years.

Pelvic ultrasound was trans-abdominally performed by a group of paediatric evaluation expert radiologists. It was used a Convex ecotomograph with B-mode ultrasound signal and a variable wavelength between 3.5 and 5.0 MHz (Philips). The subject was examined in a supine position with distended bladder and we’re detected uterine measurements [longitudinal diameters (LD), tranverse diameter (DT) and antero-posterior diameter (APD)] and ovarian measurements. It was also evaluated the visibility of the endometrial rhyme. The uterine volumes were obtained through the ellipsoid formula [(DL x DT x ADP x 0.52)/1000]. A uterine longitudinal diameters (ULD) > 34 mm and/or a uterine volume (UV) > 4 ml and/or the presence of endometrial rhyme were considered suggestive of estrogenic stimulation and advanced pubertal development (20).

### Statistical analysis

We performed a descriptive analysis of central trend measures (mean and median), with range maximum and minimum value and standard deviation of continuous variables (age at diagnosis, SDS height, SDS BMI, Tanner stage, neonatal weight, rate of progression of pubertal development, basal and peak value of LH and FSH, estradiol, bone age, uterine volume and longitudinal diameter of uterus).

We analysed Tanner stage with frequency tables. We analysed data with Chi squared tests.

We realised frequency tables for distribution analysis of descriptive variables (presence of endometrial rhyme, Tanner stage and medical therapy).

We used Student’s t-distribution tests to evaluate differences between means of the following variables, in the different groups defined on the basis of diagnosis: SDS weight, SDS height, SDS BMI, birth weight, rate of progression of pubertal development, age at diagnosis, basal and peak value of LH and FSH, bone age, uterine volume, longitudinal diameter uterus.

We used non-parametric tests (Mann–Whitney) for data with non-normal distribution and two proportion zeta tests and chi squared tests for frequency analysis.

All statistical tests were two-tailed and P < 0.05 was considered statistically significant.

Statistical analyses were performed with the use of NCSS 2021 (NCSS 2021 Statistical Software. NCSS, LLC. Kaysville, Utah, USA, ncss.com/software/ncss, 2020) for statistical analyses.

## Results

**Table 1** shows the frequency of the different diagnoses in the two periods of observation. Compared to Period 1, the Group CPP only increased significantly the frequency of diagnosis in Period 2.

**Table 1:** Frequency of different diagnoses of precocious puberty in Period 1 and Period 2.

	N° of patients	Period 1 (N%)	Period 2 (N%)	p value
CPP	26	9 (35%)	17 (75%)	**<0.05**
EP	67	28 (42%)	39 (48%)	NS
Pubarche	38	20 (52%)	18 (48%)	NS
Telarche	23	10 (43%)	13 (47%)	NS

NS, not statistically significant.

**Table 2** shows auxological data at diagnosis and rate of pubertal progression in girls with CPP diagnosed in period 2, compared with girls with CPP diagnosed in period 1. We did not find any significant difference in the rate of pubertal progression. As regards isolated pubarche, our data show a lower onset age in the pre-Covid group with statistical significance (6.75 ± 0.04 years vs 7.33 ± 0.2 years, p < 0.005), probably due to the diagnostic delay during the pandemic.

**Table 2:** Auxological data at diagnosis and rate of pubertal progression in girls with CPP subdivided by observation period.

	Period 1 (9 cases)	Period 2 (17 cases)
Age at onset (yrs, M ± SD )	6.96 ± 0.55	6.43 ± 1.5
Age at diagnosis (yrs, M ± SD )	7.27 ± 0.6	6.99 ± 1.38
Weight SDS (M ± SD)	0.93 ± 0.95	0.83 ± 0.85
Height SDS (M ± SD)	1.25 ± 1.15	1.10 ± 1.30
BMI SDS (M ± SD)	0.57 ± 0.92	0.66 ± 0.6
SDS target height (M ± SD)	-0.5 ± 0.51	0.57 ± 1.3
Rate of pubertal progression (months)	6.5 ± 2.12	4 ± 2.83
EO-EC (years, months)	1.89 ± 1.20	1.64 ± 1.16

**Table 3** shows hormonal data at diagnosis in girls with CPP subdivided by observation period. We did not find any difference in the two groups.

**Table 3:** Hormonal data at diagnosis in girls with CPP subdivided by observation period.

	Period 1 (9 cases)	Period 2 (17 cases)
Basal LH (pg/mL) (M ± SD)	2.5 ± 3.92	1.15 ± 0.64
Peak LH (M ± SD)	7.37 ± 5.03	11.42 ± 13.76
E2 > 15 pg/mL (% cases)	50%	69%

**Table 4** shows US data in girls with CPP subdivided by observation period. The percentage of girls with endometrial rhyme at diagnosis was higher in period 2, compared to period 1, but this difference was not significant, probably due to the small sample of patients.

**Table 4:** Ultrasound (US) data at diagnosis in girls with CPP subdivided by observation period.

	Period 1 (9 cases)	Period 2 (17 cases)
Uterus D.L. (M ± SD)	40.38 ± 10.78	39.53 ± 7.32
Uterine volume (M ± SD)	5.21 ± 3.63	3.88 ± 2.10
Endometrial rhyme % cases	12.5%	40%

We found 3 girls with CPP and 14 girls with EP in sub-Period 2.1 and 14 girls with CPP and 34 girls with EP in sub-Period 2.2. The comparative analysis of sub-Period 2.1 and 2.2 did not show any differences in auxological, laboratory and radiologic data except for BMI SDS that showed a significant increase in the period after the restrictive lockdown in the EP group (sub-Period 2.1: -0.73 ± 1.39, sub-Period 2.2 0.26 ± 0.72). We did not find any significance in the group with CPP, probably due to the sample’s reduced size (sub-Period 1 0.07 ± 0.79 vs sub-Period 2 0.79 ± 0.53).

In regard to the results of the questionnaire on the use of electronic devices in girls with CPP, the percentage of girls with CPP that used PCs and tablets more than 2 hours a day was significantly higher during the lockdown period, compared with the preceding period (PC 85.5% vs 0%, Tablet 15% vs 0, p < 0.005). In addition, the percentage of girls that used PCs and smartphones more than 2 hours a day during lockdown was significantly higher in girls with CPP compared with the control group (PC 85.5% vs 73%, p < 0.005, Smartphone 29% vs 10% p < 0.005).

## Discussion

CPP is a rare disease that involves mainly females and in most cases it is idiopathic.

The results of our study, in line with other Italian studies recently published (8–11), show an increase in the number of new diagnoses of CPP during lockdown and in the following months, compared with previous period.

Moreover, in accordance with the results of Chioma et al (10), this increase is observed only in patients with the classical form of CPP, with onset before 8 years of age, while it was not observed in girls with isolated forms of pubarche and thelarche. These results therefore prompt the hypothesis that the factors potentially responsible for this increase specifically induce the precocious activation of hypothalamic-pituitary axis.

During the SARS-CoV-2 pandemic, people’s habits changed radically; we think that an environmental change in lifestyle may influence pubertal timing.

We assumed that obesity and the increase of BMI in the paediatric population are possible contributing factors of the pubertal onset advance seen in the last few decades in industrialized countries. The increase in fat mass may lead to an augmented production of adipocyte hormones like leptin, which has a permissive role in HPG axis activation (21) (22), and insulin, which promotes GnRH activity, mostly mediated by leptin (23) (24). The increased weight leads to augmented hypothalamic ceramide levels, a lipid signalling molecule with a permissive role on the HPG axis (25) and lead to a reduction of Sirtuin 1, a deacetylase that negatively influences pubertal activation.

In our experience, we found no significant increase in SDS BMI in girls with CPP diagnosed in Period 2, but only a trend of increasing BMI in months after lockdown in girls with EP. Indeed only in Stagi’s study (8) was an increase observed in BMI in girls with CPP diagnosed during lockdown, while no other Italian studies detected this change. On the other hand, we have to consider the reduction of physical activity that characterized the lockdown and the following months, with the persistence of distance learning in the paediatric population. This lack of physical activity may have had a negative influence on body composition resulting in an increase in fat mass without an increase in BMI (10).

In regard to a greater pubertal thrust that characterized the diagnosis of forms of CPP detected during lockdown, as compared with forms identified in the previous period, there is contrasting literature data.

The studies of Stagi (8) and Umano (11) report levels of gonadotropins significantly higher in girls with CPP diagnosed during lockdown compared with the previous period. Our data, according to the multicentre study of Chioma et al (10), show no differences in hormone levels at diagnosis.

In Stagi’s (8) study the number of girls with CPP who experienced a transition from slowly progressive pubertal development to accelerated pubertal development during lockdown was significantly higher compared with the previous 5 years. We did not observe this increase in pubertal progression, although our evaluation was based on referred data and not on clinical evaluation.

On the other hand, as concerns US data, our study is the only one in literature that shows a higher percentage of girls with the presence of endometrial rhyme during lockdown, compared with previous period.

It is well known that the presence of endometrial rhyme is a really specific parameter of estrogenic secretion and significant pubertal activation (24). It could be assumed that the estrogenic boost was more intensive in patients with onset during lockdown and that this favoured the onset of endometrial rhyme, despite the similarity of laboratory data.

We must however considerer that controversial results in the literature could depend on the difficulty of comparing studies having different numbers of patients, inclusion criteria and observation period.

The lifestyle change due to lockdown led to an increase in the use of electronic devices. The use of PCs increased more than other devices, due to distance learning. We detected that patients with CPP used more PCs and smartphones during lockdown, compared to the control group. Some studies assume that the electromagnetic radiation produced by electronic devices causes a down regulation in melatonin secretion (6, 7, 26). Normally, melatonin levels decrease during puberty and this down regulation leads to an activation of GnRH *via* genetic transcription (27). The increased use of electronic devices could have determined a melatonin level reduction that may have favoured the development of precocious puberty in susceptible individuals. This data may justify the stability of other forms of non-central precocious puberty like EP, isolated pubarche and thelarche.

Psychological factors linked to the pandemic are other environmental factors that we may consider. Some studies showed an increase of stress factors (fear of disease, drastic change of habits, break up of social contacts) (8, 9). Stress factors lead to an increase and then a decrease of cortisol levels. Cortisol has a negative feedback on the HPG axis; we can assume that a decrease in cortisol could have favoured the development of puberty. The examination of psychological effects is outside our analysis but we can assume there was indeed an increase in stress factors in our patients.

### Strengths

We performed all the evaluations in the same Centre with homogeneous criteria of diagnosis and evaluation.

We also analysed the frequency of other forms of precocious puberty without central involvement, in contrast to other published studies.

We used a control group for the examination of the questionnaires regarding the use of electronic devices.

### Limits

The limitations of our study are largely related to the emergency situation due to the pandemic, which did not allow prospective, controlled studies.

The sample small size and restrospective nature are also in the limitations of our study.

The data are preliminary and other evaluations need to be carried out through multicentre analyses with wider case studies.

## Conclusion

The SARS-CoV-2 pandemic led to radical habit changes and we assume that these changes influenced the increase in frequency of CPP. Based on our data we assume there was an environmental effect on pubertal timing that calls our attention to factors such as food, use of electronic devices and stress. We will need other studies to better understand this data. By improving our knowledge of these factors, we could better understand some of the physiopathological aspects of puberty and implement preventive and corrective actions.

## Data availability statement

The original contributions presented in the study are included in the article/supplementary material. Further inquiries can be directed to the corresponding author.

## Ethics statement

The studies involving human participants were reviewed and approved by UOC Ricerca e Innovazione - Scudeller Startup/Istruttoria (CE Locale) IRCCS Azienda Ospedaliero-Universitaria di Bologna Policlinico S.Orsola-Malpighi. Written informed consent to participate in this study was provided by the participants’ legal guardian/next of kin.

## Author contributions

All authors listed have made a substantial, direct, and intellectual contribution to the work and approved it for publication.

## Conflict of interest

The authors declare that the research was conducted in the absence of any commercial or financial relationships that could be construed as a potential conflict of interest.

## Publisher’s note

All claims expressed in this article are solely those of the authors and do not necessarily represent those of their affiliated organizations, or those of the publisher, the editors and the reviewers. Any product that may be evaluated in this article, or claim that may be made by its manufacturer, is not guaranteed or endorsed by the publisher.

## References

[1] Roberts SA Kaiser UB. Genetics in endocrinology genetic etiologies of central precocious puberty and the role of imprinted genes. Eur J Endocrinol (2020) 183:R107–17. doi: 10.1530/EJE-20-0103 PMC7682746 32698138

[2] Abreu AP Kaiser UB. Pubertal development and regulation. Lancet Diabetes Endocrinol (2016) 4(3):254–64. doi: 10.1016/S2213-8587(15)00418-0 PMC5192018 26852256

[3] Maione L Bouvattier C Kaiser UB. Central precocious puberty: recent advances in understanding the aetiology and clinical approach. Clin Endocrinol (2021) 95:542–55. doi: 10.1111/cen.14475 PMC8586890 33797780

[4] Brix N Ernst A Lauridsen LLB Parner ET Arah OA Olsen J. Childhood overweight and obesity and timing of puberty in boys and girls: Cohort and sibling-matched analyses. Int J Epidemiol (2021) 49:834–44. doi: 10.1093/ije/dyaa056 PMC7394964 32372073

[5] Amigo H Vásquez S Bustos P Ortiz G Lara M. Socioeconomic status and age at menarche in indigenous and non-indigenous Chilean adolescents. Cad Saude Publica (2012) 28:977–83. doi: 10.1590/S0102-311X2012000500016 22641520

[6] Waldhauser F Weiszenbacher G Tatzer E Gisinger B Waldhauser M Schemper M. Alterations in nocturnal serum melatonin levels in humans with growth and aging. J Clin Endocrinol Metab (1988) 66(3):648 –52. doi: 10.1210/jcem-66-3-648 3350912

[7] Waldhauser F Waldhauser M Lieberman HR Deng MH Lynch HJ Wurtman RJ. Bioavailability of oral melatonin in humans. Neuroendocrinology (1984) 39(4):307–13. doi: 10.1159/000123997 6493445

[8] Stagi S De Masi S Bencini E Losi S Paci S Parpagnoli M. Increased incidence of precocious and accelerated puberty in females during and after the Italian lockdown for the coronavirus 2019 (COVID-19) pandemic. Ital J Pediatr (2020) 46(1):165. doi: 10.1186/s13052-020-00931-3 33148304 PMC7609833

[9] Verzani M Bizzarri C Chioma L Bottaro G Pedicelli S Cappa M. Impact of COVID-19 pandemic lockdown on early onset of puberty: Experience of an Italian tertiary center. Ital J Pediatr (2021) 47(1):52. doi: 10.1186/s13052-021-01015-6 33673836 PMC7935003

[10] Chioma L Bizzarri C Verzani M Fava D Salerno M Capalbo D. Sedentary lifestyle and precocious puberty in girls during the COVID-19 pandemic: an Italian experience. Endocr Connect (2022) 11(2):e210650. doi: 10.1530/EC-21-0650 35029543 PMC8859940

[11] Umano GR Rondinelli G Rivetti G Klain A Aiello F Miraglia Del Giudice M. Effect of covid – 19 lockdown on children’s eating behaviours: A longitudinal study. Children (Basel) (2022) 20:9. doi: 10.3390/children9071078 PMC9323163 35884062

[12] Carel JC Eugster EA Rogol A Ghizzoni L Palmert MR ESPE-LWPES GnRH Analogs Consensus Conference Group. Consensus statement on the use of gonadotropin-releasing hormone analogs in children. Pediatrics (2009) 123(4):e752-62. doi: 10.1542/peds.2008-1783 19332438

[13] Mul D Oostdijk W Drop SL. Early puberty in girls. Best Pract Res Clin Endocrinol Metab (2002) 16(1):153–63. doi: 10.1053/beem.2001.0187 11987905

[14] Cacciari E Milani S Balsamo A Spada E Bona G Cavallo L. Italian Cross-sectional growth charts for height, weight and BMI (2 to 20 yr). J Endocrinol Invest (2006) 29:581–93. doi: 10.1007/BF03344156 16957405

[15] Marshall WA Tanner JM. Variations in pattern of pubertal changes in girls. Arch Dis Child (1969) 44:291–303. doi: 10.1136/adc.44.235.291 5785179 PMC2020314

[16] Carel JC Leger J. Clinical practice. precocious puberty. N Engl J Med (2008) 358:2366–77. doi: 10.1056/NEJMcp0800459 18509122

[17] Neely EK Hintz RL Wilson DM Lee PA Gautier T Argente J. Normal ranges for immunochemiluminometric gonadotropin assays. J Pediatr (1995) 127:40–6. doi: 10.1016/s0022-3476(95)70254-7 7608809

[18] Bangalore Krishna K Fuqua JS Rogol AD Klein KO Popovic J Houk CP. Use of gonadotropin-releasing hormone analogs in children: update by an international consortium. Horm Res Paediatr (2019) 91:357–72. doi: 10.1159/000501336 31319416

[19] Greulich WW Pyle SI. Radiographic atlas of skeletal development of the hand and wrist. Calif Med (1959) 91(1):53.

[20] Orsini LF Salardi S Pilu G Bovicelli L Cacciari E. Pelvic organs in premenarcheal girls: Real- time ultrasonography. Radiology (1984) 153(1):113–6. doi: 10.1148/radiology.153.1.6473771 6473771

[21] Ahima RS Saper CB Flier JS Elmquist JK. Front leptin regulation of neuroendocrine systems. Neuroendocrinol (2000) 21(3):263–307. doi: 10.1006/frne.2000.0197 10882542

[22] Considine RV Sinha MK Heiman ML Kriauciunas A Stephens TW Nyce MR. Serum immunoreactiveleptin concentrations in normal-weight and obese humans. N Engl J Med (1996) 334(5):292–5. doi: 10.1056/NEJM199602013340503 8532024

[23] Moret M Stettler R Rodieux F Gaillard RC Waeber G Wirthner D. Insulin modulation of luteinizing hormone secretion in normal female volunteers and lean polycystic ovary syndrome patients. Neuroendocrinology (2009) 89(2):131–9. doi: 10.1159/000160911 18832802

[24] Barr VA Malide D Zarnowski MJ Taylor SI Cushman SW. Insulin stimulates both leptin secretion and production by rat white adipose tissue. Endocrinology (1997) 138(10):4463–72. doi: 10.1210/endo.138.10.5451 9322964

[25] Heras V Castellano JM Fernandois D Velasco I Rodriguez-Vasquez E Roa J. Central ceramide signaling mediates obesity-induced precocious puberty. Cell Metab (2020) 32:951–966.e8. doi: 10.1016/j.cmet.2020.10.001 33080217

[26] Lewczuk B Redlarski G Zak A Zió łkowska N Przybylska-Gornowicz B Krawczuk M. Influence of electric, magnetic, and electromagnetic fields on the circadian system: Current stage of knowledge. BioMed Res Int (2014) 2014:169459. doi: 10.1155/2014/169459 25136557 PMC4130204

[27] Roy D Belsham DD. Melatonin receptor activation regulates GnRH gene expression and secretion in GT1-7 GnRH neurons. Signal transduction mechanisms. J Biol Ch (2002) 277(1):251–8. doi: 10.1074/jbc.M108890200 11684691

